# Special Attention to Diet and Physical Activity in Children and Adolescents With Obesity During the Coronavirus Disease-2019 Pandemic

**DOI:** 10.3389/fped.2020.00407

**Published:** 2020-06-26

**Authors:** Valeria Calcaterra, Matteo Vandoni, Vittoria Carnevale Pellino, Hellas Cena

**Affiliations:** ^1^Pediatric and Adolescent Unit, Department of Internal Medicine, University of Pavia, Pavia, Italy; ^2^Pediatric Unit, Children's Hospital V. Buzzi, Milan, Italy; ^3^Laboratory of Adapted Motor Activity (LAMA), Department of Public Health, Experimental and Forensic Medicine, University of Pavia, Pavia, Italy; ^4^Department of Industrial Engineering, University of Tor Vergata, Rome, Italy; ^5^Laboratory of Dietetics and Clinical Nutrition, Department of Public Health, Experimental and Forensic Medicine, University of Pavia, Pavia, Italy; ^6^Clinical Nutrition and Dietetics Service, Unit of Internal Medicine and Endocrinology, ICS Maugeri IRCCS, Pavia, Italy

**Keywords:** obesity, COVID-19, children, diet, physical activity

Coronavirus disease 2019 (COVID-19) is an acute infectious respiratory disease that has posed critical challenges for the global healthcare community. Following the outbreak of COVID-19 (1), the Italian government imposed a national quarantine, restricting the movement of the population as a fundamental safety step to limit exposure to the virus and contain contagion. All schools were closed, requiring childcare and education to be provided at home; public spaces were also closed, and mobility was restricted to health or work situations. Unfortunately, the mandatory directives locking down outdoor activities inevitably disrupted the daily routine of children, including regular physical activity and excercise. This increased the risk of major weight gain for children already prone to gaining weight. Therefore, eating healthy foods and being physically active is recommended.

COVID-19 involves all age groups, although children are less likely to develop severe illness than adults (2). In adults, conditions such as chronic lung diseases, hypertension, cardiovascular disease, and diabetes, seem to increase the risk for an adverse COVID-19 outcome. The effect of obesity on the outcome remains controversial. Initially, such implications were not seriously considered; however, recent papers showed an association between obesity and severe outcome (3).

So far no reports on severity of disease in children with obesity compared to normal-weight subjects have been reported, that we know of. However, several studies show that obesity is associated with inflammation and severe airway obstruction in patients with respiratory tract infections (4). As reported by Okubo (5), pediatric obesity is an independent risk factor for severity and morbidity among children with lower respiratory tract infections by means of potential factors including subclinical inflammation, obesity-related immune system dysregulation, decreased cell-mediated immune responses, and obesity-related respiratory dysfunction (6). Adipose tissue expresses components of the renin-angiotensin system (RAS) (7), including the expression of angiotensin-converting enzyme 2 (ACE2–the functional receptor for SARS-CoV), which is up-regulated in the adipocytes of subjects with obesity, turning adipose tissue into a potential target and viral reservoir. Additionally, in high-fat-fed animal experimental models, researchers described dysregulated ACE2 expression as increasing the risk of COVID-19 infection (7).

Lockdowns may worsen not only the weight but also the eating habits of children, since homes are likely stocked with ultra-processed and calorie-dense comfort foods (8). Good nutrition is very important before, during, and after an infection. Although COVID-19 infection cannot be prevented by any food or dietary supplements, maintaining a healthy diet is an important part of supporting a strong immune system (4, 8).

Diet and nutrition play an important role in inflammation and immunity. Specific foods (8), including simple sugars, trans fats, refined carbohydrates, and processed meat, may promote inflammation and also counteract the anti-inflammatory effects of omega-3 fatty acids (9). Therefore, consumption of junk food may increase systemic inflammation in subjects with overweight or obesity, promoting IL-6 production (4).

Although inflammation is one of the body's first responses to infection, overactive immune responses in a persistent stress and inflammation condition may increase risk of severe infections.

Keeping children on a healthy diet in a safe home environment is an important strategy for maintaining weight control for children with obesity during this emergency coronavirus social lockdown, as is promoting physical activity (Table 1).

Table 1Crucial advice for diet and physical activity in children and adolescents with obesity during the COVID-19 pandemic.

**Table:** 

**Eating habits and behaviors**	**Anti-inflammatory foods and hydration**
**DIET**	
1. Sit down to family meals	1. Vegetables: *rich in Vitamin C (eg: citrus fruits, berries, bell peppers, broccoli, and sweet potatoes); rich in Poliphenols (eg: berries, apples, beans, nuts, artichokes, spinach); rich in prebiotics*
2. Three meals and two snacks a day	2. Fruit: *rich in Vitamin C (eg: citrus fruits, berries, bell peppers, broccoli, and sweet potatoes); rich in Poliphenols (eg: berries, apples, beans, nuts, artichokes, spinach); rich in prebiotics*
3. Varied diet (five food groups per meal)	3. Yogurt: *rich in probiotics*
4. Make half of your plate vegetables and fruits	4. Fish: *rich in omega 3*
5. Keep healthy food at hand	5. Nuts: *rich in Poliphenols (eg: berries, apples, beans, nuts, artichokes, spinach)*
6. Prepare dishes in the kitchen together	6. Whole grains: *rich in prebiotics*
7. Correct portion sizes	7. Dried fruits and vegetables: *rich in Poliphenols (eg: berries, apples, beans, nuts, artichokes, spinach); rich in prebiotics*
8. Teach children to make healthy desserts with fruit and yogurt	8. Beans: *rich in Poliphenols (eg: berries, apples, beans, nuts, artichokes, spinach)*
9. Never use food as a reward	9. Olive oil: *rich in Poliphenols (eg: berries, apples, beans, nuts, artichokes, spinach)*
10. Give children some control	10. Water

**Table:** 

**Ranking**	**Name of the game**	**Ability**	**How to play**	**Duration**	**Frequency**
**PHYSICAL ACTIVITY**					
1	Active breaks	Metabolism enhancement	Choose an exercise or a combination and do it non-stop	2–3 min	Every hour
2	Clean-up race	Agility and coordination	Set a timer or put on a song to see who can put the room to rights fastest, or clean the kitchen	Fastest!	Once a day
3	Play with pets	Aerobic training	Walk, run, jump, and play with balls with pets	20 min	Two or three times a day
4	Balloon games	Metabolism enhancement	Play with a balloon in a different way	30 min	Four times a week
5	Musical party	Aerobic training	Play music and dance or imitate “stars” on TV	30 min	Two times a week
6	Animal races	Coordination and resistance	Move like an animal (frog, crayfish, penguin, snake, etc.)	20 min	Four times a week
7	Obstacle course	Agility and coordination	Create an obstacle course with furniture in your apartment or outside.	30 min	Three times a week
8	Tape game	Coordination and resistance	Create shapes on the floor with tape and give instruction to complete a path.	30 min	Two times a week
9	Follow the leader	Metabolism enhancement	Focus on a sport, an activity, or an action. Imitate a person who has the leader role. Can also be played in video chat.	30 min	Two times a week
10	Exergames	Mixed	Play active videogames	An hour	Three times a week
1) Choose a few simple exercises: Walk on the spot, stretch arms and legs out to the side like a starfish while jumping, return arms to sides and legs to center on landing, circle arms, etc.					
2) Parents' satisfaction: Children could help parents in the housework as an active play activity.					
3) Play with pets: Walk or run in the house or outside, creating small paths.					
4) Balloon games: Alone or with parents, children could throw the balloon at a wall, bounce it over their head, dribble it on a chair or table, hit it up in the air but do not let it touch the ground, place it between their knees and waddle across the room without dropping it.					
5) Musical party: Sing and dance imitating a video on the internet.					
6) Animal races: Children walk, hop, or crawl in the styles of various animals.					
7) Obstacle course: Create an obstacle course with furniture in an apartment or outside. Some tools could be added: hula hoops to jump through, a line of tape to balance on, a table to crawl under, a blanket over two chairs to crab-walk through, etc.					
8) Tape game: Parents use tape to lay a variety of shapes, letters, and/or numbers on the floor and prepare instructions to follow, e.g., “bear crawl to the square,” “hop like a frog to the T,” or “run to the rectangle.”					
9) Follow the leader: Stand face to face, about a foot apart, and have the child attempt to copy all your movements, reach up and stretch to the sky, do 10 jumping jacks, act like a monkey, etc.					
10) Exergames: Use technology that uses interactive games to increase exercise behavior by requiring the players to physically interact with onscreen avatars through a variety of body movements while providing players the opportunity of being physically active and promoting their overall health.					

Children need to play and keep physically active to protect their physical and emotional health during growth (10). In particular, physical activity (PA) contributes to daily energy expenditure, thus increasing lean body mass, improving energy intake and metabolic and psychological profiles (10). A previous study by McManus et al. (11) on PA evaluation in normal weight and obese children showed no difference between children regarding moderate to vigorous PA, but further analysis showed that lack of light-intensity tasks in obese children explained the difference in total daily energy expenditure. Thus, in obese children, acquiring correct PA targets by means of frequent, short-duration day-to-day tasks, rather than sustained organized sport or exercise, is crucial. In fact, since obese children do not usually spend their leisure time in light-intensity activities, we believe that proper suggestions of games and active lifestyle habits will be crucial in confinement to small spaces. During the COVID-19 pandemic, PA or exercise restriction at school or in outdoor settings leads to a vicious cycle of sedentary behavior and decreased daily energy expenditure ending in weight gain. In light of this, the implementation of recreation and games as well as programmed PA at home becomes of primary importance.

In order to promote adherence to PA, we suggest different games (Table 1) that should be chosen according to the characteristics and personal preferences of the child. For each activity and game, recommendations are made for the duration and intensity necessary to gain muscular strength and flexibility, to improve fundamental motor skills and functions such as cardiorespiratory endurance, core stability, balance, and posture, and also to have fun.

Healthy diet and behaviors such as programmed physical activity, limited screen time, and adequate sleep may help children deal with these required social restriction rules, contributing to positive emotions, emotional stress responses, weight control, and health.

## Author Contributions

VC, MV, VP, and HC made a substantial contribution to the concept or design of the work, drafted the article, or revised it critically for important intellectual content. All authors approved the version to be published.

## Conflict of Interest

The authors declare that the research was conducted in the absence of any commercial or financial relationships that could be construed as a potential conflict of interest.
